# Screening Older Adults for Hearing Loss in Primary Care: Insights of Patients, Providers, and Staff

**DOI:** 10.1093/geroni/igaa057.3359

**Published:** 2020-12-16

**Authors:** Mina Silberberg, Anisha Singh, Janet Bettger, Rowena Dolor, Sherri Smith, Howard Francis

**Affiliations:** 1 Duke University, Durham, North Carolina, United States; 2 Duke University School of Medicine, Durham, North Carolina, United States; 3 Duke University Medical Center, Durham, North Carolina, United States

## Abstract

Over one-third of older adults have a disabling hearing loss, with potentially severe implications for well-being. Hearing screening is not routine in primary care (PC) and patients are relied upon to report hearing concerns. We compared outcomes of three approaches to linking telephone-based screening with PC (providing information at PC visit, encouraging at visit, or completing at visit). This poster presents results of focus groups/interviews with providers and staff from participating clinics (n= 35), study enrollees who completed screening and were referred for diagnosis (n=14 ), and enrollees who did not complete screening (n=12). Results show that most patients had prior hearing concerns they had not reported to their PC. Patients forgot or were resistant to completing screening at home. Negative attitudes towards admitting hearing loss and using hearing aids were common; experiences of family and friends influenced many patient attitudes, both negative and positive. PC personnel wish to help, but are challenged by lack of time, space, and reimbursement for screening, and loathe to screen when specialty care and hearing aids are costly. Study results indicate that relying on patients to report hearing concerns is inadequate. Integration of hearing screening into PC would be helped by strengthening reimbursement for screening, specialty care, and hearing aids, and education of both providers and patients on other available treatments for hearing loss. Patients also require education on hearing aid technology. There is a need to address stigma associated with hearing loss, taking into consideration the influence of family and friends on attitudes.

